# Staff Awareness of Anti-Cholinergic Burden (ACB) - A Qualitative Cross-Sectional Study in a Tertiary Care Hospital

**DOI:** 10.7759/cureus.14141

**Published:** 2021-03-27

**Authors:** Kamalaveni Soundararajan, Pooja Balchandra

**Affiliations:** 1 Obstetrics and Gynaecology, Hull University Teaching Hospitals NHS Trust, Kingston upon Hull, GBR

**Keywords:** anti cholinergic burden, acb, awareness, health care staff

## Abstract

Introduction and hypothesis

Anticholinergics are commonly used for a variety of conditions including urinary incontinence. Many studies show the ill effects of anticholinergics on cognition resulting in increased morbidity and mortality. However, the interaction of anticholinergic medications and cumulative anti-cholinergic burden (ACB) of different medications are not well known in general population and amongst health care professionals. Our aim is to study the extent of current awareness of ACB amongst health care professionals which plays a crucial role in educating patients and avoiding these morbidities.

Methods

A single centre cross-sectional study of 50 health care professionals who participated voluntarily. A questionnaire was designed to assess the knowledge, beliefs and attitudes towards anticholinergic burden and participants were also asked to choose the ACB score for 17 commonly used medications.

Results

A total of 74% participants admitted to have no understanding of the term ACB, 48% participants prescribe anticholinergics in their daily role, 44% knew that cognition was adversely affected by anticholinergics, and 16% participants were aware of scoring system. Only 16% participants routinely counsel women of cognitive side effects when anticholinergics are started. 86% reported that they would avoid prescribing medications which might affect cognition if possible. If given choice as a patient, 94% would avoid these medications if they were informed of the specific side effects like impaired cognition, physical decline, falls, hospital admissions and increased mortality.

Conclusion

Anticholinergic burden (ACB) is a serious phenomenon associated with increased morbidity and mortality in the general population as well as elderly population. It is evident from this study that the knowledge and awareness of ACB in our health care staff are still lacking.

## Introduction

Anticholinergics are commonly used to treat not only urinary incontinence due to overactive bladder (OAB) but also chronic obstructive pulmonary disease (COPD), asthma, Parkinson’s disease, dizziness and motion sickness. Many studies show the ill effects of anticholinergics on cognition resulting in increased morbidity and mortality [1, 2]. However, the interaction of anticholinergic medications and cumulative anti-cholinergic burden (ACB) of different medications are not well known in general population and amongst health care professionals [3]. The drugs used for OAB are competitive inhibitors of muscarinic anticholinergic receptors in the bladder [4]. Common side effects include dry mouth, blurred vision, constipation and drowsiness as well as head ache, nausea, palpitations, tachycardia, urinary disorders and vomiting [4,5]. Confusion and angioedema are given as rare or very rare side effects in the British National Formulary [5]. There are also other medications which can have anticholinergic effects and using two or more of these medications together results in cumulative ACB. There are many recent studies revealing cognitive impairment with anticholinergic burden in people over the age of 50-65 [6, 7-12]. There seems to be a dose-response relationship between anticholinergic use and increased risk of dementia [6, 12]. In addition to dementia, there are also increased risks of mortality associated with ACB [13]. Our aim was to study the extent of current awareness of health care professionals of ACB which plays a crucial role in educating patients and avoiding these morbidities.

## Materials and methods

We designed a qualitative cross-sectional study to assess the knowledge, beliefs and attitudes of health care staff towards anticholinergic burden. Participants were also asked to choose the ACB score for 17 commonly used medications (Table 1). We used a purpose made questionnaire (Appendix, Table 2). All health care professionals in our unit were given the opportunity to participate and complete the questionnaire on a voluntary basis over the course of one week from 1st October 2020. The short interval was used to minimize peer group bias and to obtain genuine personal views. Fifty staff participated and returned the questionnaire. Data was analysed with the help of Microsoft Excel. The free text answers were analysed by manual review of each response and information was collated.

**Table 1:** Anti-cholinergic burden (ACB) scores of the medications

Drug with ACB 0	Drugs with ACB 1	Drugs with ACB 2	Drugs with ACB 3
Mirabegron	Tramadol	Cetirizine	Fesoterodine
	Hydrocortisone		Tolterodine
	Prednisolone		Darifenacin
	Codeine		Trospium
	Warfarin		Oxybutynin
	Nifedipine		Chorpheneramine
	Hydralazine		Promethazine
			Amitriptyline

## Results

Of the total 50 participants (n = 50), there were 17 nursing and allied health care staff and 33 were doctors. There were seven auxiliary nurses, two midwives, eight nurses, three general practice trainee doctors, five obstetrics and gynecology specialty trainees, 13 obstetrics and gynecology specialty registrars and 12 consultants. Only 38% (n = 19) of staff were aware of the term anti-cholinergic burden; 74% (n = 37) staff admitted to have no understanding of the term ACB; however, 48% (n = 24) participants prescribed anticholinergics in their daily role, 44% (n = 22) staff knew that cognition was adversely affected by anticholinergics.

Overall, 16% (n = 8) participants were aware of scoring system for cognitive burden, none of them were nursing or allied health care staff. Only 24% (n = 8) of medical staff were aware of the scoring system for ACB; 50% of first on call doctors, 15% of second on call doctors and 17% of consultants were aware of a scoring system for ACB. The ACB scores of the 17 medications used in the questionnaire are given in Table 1.

**Figure 1:**
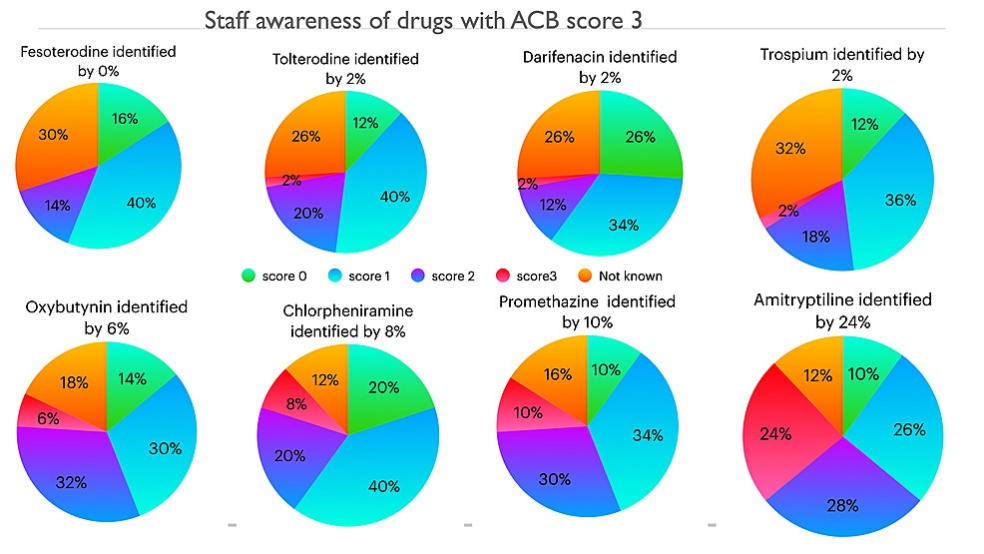
Staff awareness of drugs with ACB score 3 ACB: Anticholinergic Burden

**Figure 2:**
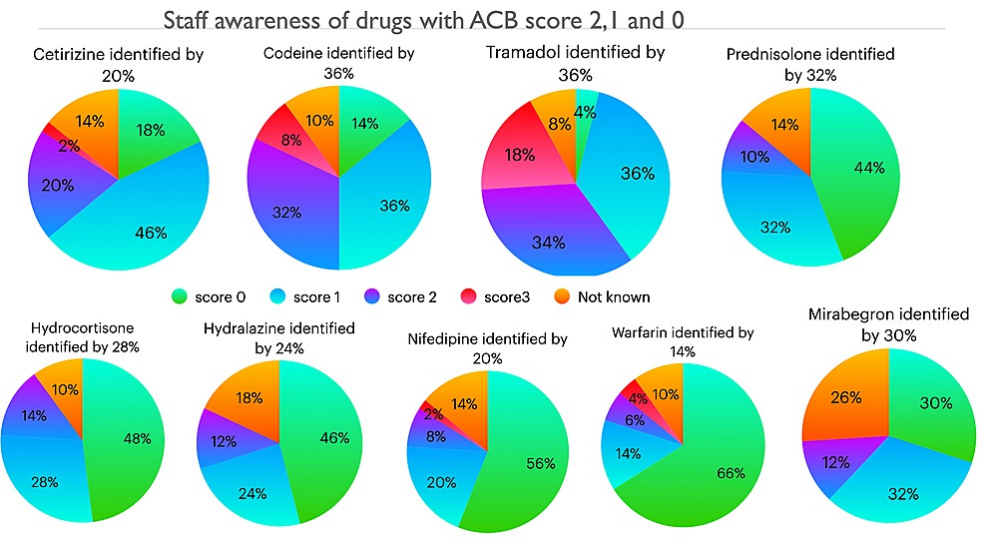
Staff awareness of drugs with ACB score 2, 1 and 0 ACB: Anticholinergic Burden

**Figure 3:**
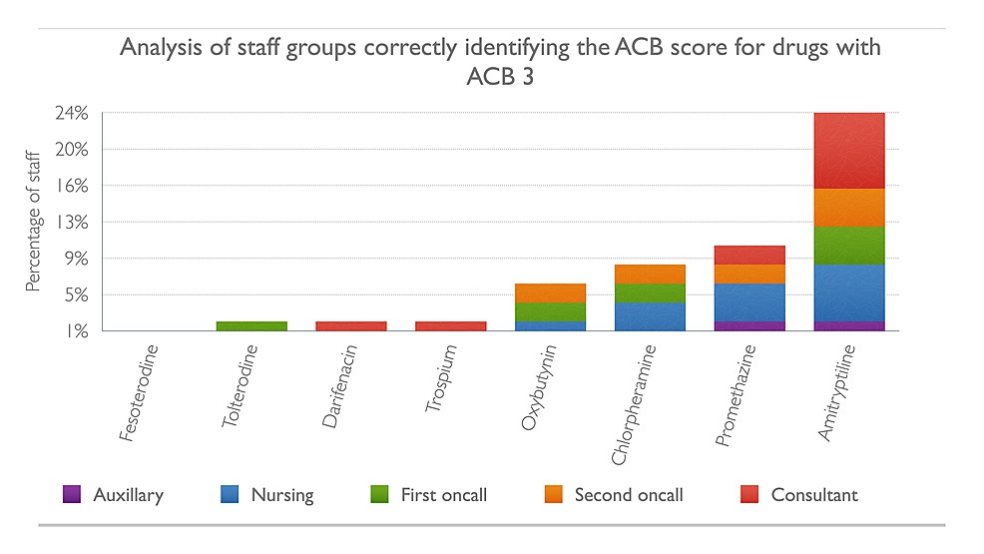
Staff groups correctly identifying ACB score of drugs with ACB 3 ACB: Anticholinergic Burden

**Figure 4:**
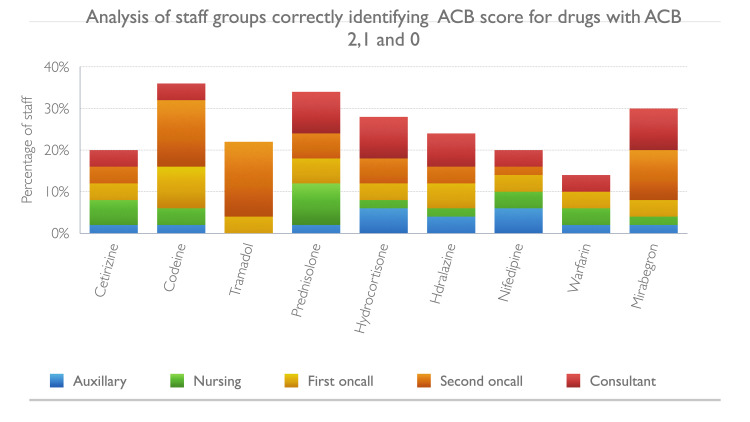
Staff groups correctly identifying ACB score for drugs with ACB 2, 1 and 0 ACB: Anticholinergic Burden

The results of staff scoring for 17 commonly used medications are given in Figures 1-4. Between 12% and 26% staff did not want to guess the score of one or more of these medications and answered not known. Up to 10% staff correctly identified the ACB score of 3 for the following medications: fesoterodine, tolterodine, darifenacin, trospium, oxybutynin, chlorpheniramine and promethazine. Amitriptyline which has a ACB score of 3 was better known among staff compared to other drugs with ACB 3, as 24% correctly identified its score. Cetirizine, a commonly used antihistamine, has a ACB score of 2 which was correctly identified by 20% staff and 14% did not know the score, 18% thought it had no anticholinergic burden and 46% thought it had mild ACB score of 1. For the drugs with mild ACB score of 1, between 14-36% staff correctly identified the score and however 4-56% failed to appreciate the cognitive burden associated with these drugs; between 8-10% staff were not sure of the score.

Eighty-six percent (n = 43) reported that they would avoid prescribing medications which might affect cognition if possible. Only 16% (n = 8) routinely counsel women of cognitive side effects when anticholinergics are started. If given choice as a patient, 94% (n = 47) would avoid these medications if they were informed of the specific side effects like impaired cognition, physical decline, falls, hospital admissions and increased mortality.

When asked what they mean by the term ACB, we received the following replies.

Responses from first on call doctors are as follows:

“Severity of anticholinergic symptoms with multiple medications” “Increased side effects of anticholinergic medications” “Side effects started by anticholinergics” “Overall effect of using different medications that contain anticholinergic properties”

Responses from second on call doctors are as follows:

“Patients on polypharmacy on multiple medications might have >1 drug with anticholinergic effect increasing the risk of untoward side effects”. “Extra pyramidal side effects” “Use of anticholinergic drugs in high enough doses to cause extra pyramidal side effects” “Side effects of dry mouth, nausea and risk of falls” “Older adults have anticholinergic side effects from polypharmacy. “Side effects outweigh benefits”

Two Consultants replied in free text:

“Synergistic effects of drugs leading to cognitive dysfunction, mainly from anticholinergic.” “Patient on multiple anticholinergics at risk of side effects.”

It is evident from above responses that the real depth of anti-cholinergic burden in relation to cognition and morbidity has not been appreciated by the staff who believed they knew about ACB.

## Discussion

Anticholinergic cognitive burden has been known for many years and scoring systems exist, however, this is not well known or well incorporated in our daily practice, despite previous study by Araklitis et al. highlighting this problem four years ago [14]. There has been no other study in literature addressing this prime issue of staff awareness in this anticholinergic burden issue and no studies available to highlight the successful interventions to increase awareness of staff and patients. Another study analysing the European Prospective Investigation into cancer (EPIC)-Norfolk general population consisting of 21,722 participants with a mean follow-up of 18 years found that ACB > 3 had 59% relative risk of incident stroke and 86% relative risk of stroke mortality compared to ACB 0 category [15]. There are various scales available to measure the anticholinergic burden of different medications like anticholinergic cognitive burden scale [16], anticholinergic risk scale [17] and online anticholinergic burden calculator [3]. We have used the online ACB calculator for ease of reference [3]. We included all genre of health care staff to study the awareness in all groups.

From reviewing the participants' comments, we understand there is assumption that ACB is only relevant to older people and side effects of progressive cognitive decline, dementia, falls are not widely appreciated; majority have indicated they had general side effects like dry mouth. Some participants wrongly believe that anticholinergic medications cause extra pyramidal side effects while anticholinergics are used to treat extra pyramidal side effects. The participants were not aware that common medications which are not graded as anticholinergics like warfarin, chlorpheniramine and cetirizine can also have cumulative anticholinergic effect.

The ACB effects are proportionate to the dose and duration of exposure. Studies involving 3707 nursing home residents over the age of 65 matched with 3707 controls, have found that risk of cognitive impairment increased with cumulative anticholinergic score ≥ 3 at 60 and 90 days of exposure. The findings remained consistent with a anticholinergic cognitive burden scale [18]. Retrospective cohort study by Campbell et al., involving a total of 3344 community dwelling older adults of age 65 and above, showed increased rate of health care utilisation in the form of inpatient, emergency department and outpatient visits as well as cognitive impairment with increasing total daily ACB score [8]. Another systematic review and meta-analysis by Ruxton et al., of 18 studies with 124,286 participants, concluded that exposure to drugs with anticholinergic effects may increase the risks of cognitive impairment, falls and all cause mortality in older adults (>=65 years) [19].

One participant, a second on call doctor, has mentioned: *“I will counsel on the anticholinergic effects depending on the age of the patient”.*

This is based on the assumption that the side effects are expected to happen only in the elderly. However, it can affect any age group. Recent studies have shown pediatric patients may also be vulnerable to anticholinergic burden mainly in the form of delirium [20, 21]. A recent systematic review and meta-analysis of Chan et al., of 46 published studies, has concluded that anticholinergic medications do not appear to detrimentally affect cognitive function in children conflicting with reviews in older adults; however, they only included 21 anticholinergics and duration of use but the dose of drug was not included in this study. All the studies were experimental and there were differences in classification of cognition in children compared to adults [22].

In a Gynecology unit, patients on overactive bladder medications are commonly managed in outpatient as well as inpatient set up. However, there was little awareness of many of these medications. A systematic review of 2122 records and meta-analysis of six studies by Dmochowski et al., on the impact of anticholinergic use for ≥3 months on dementia in adult patients, concluded that there is increased risk of dementia by 46%, which was consistent in studies assessing overactive bladder medications [23]. It is worrying to note that in our study, 44% knew about the cognitive side effects, however, only 16% counsel patients on cognitive side effects while they are started on these medications. Eighty-six percent (n = 43) participants have indicated that they would consider avoiding prescribing the medications that affects cognition if possible; this shows the knowledge gap and given the guidance and advice with easy reference, many of the health care workers would like to avoid the cognitive adverse effects. This can be achieved by use of online ACB calculator, memory aids and thorough analysis of all regular medications of the patient which will add to ACB as well as patient’s current cognitive state at the time of prescription of any anticholinergic medication. This knowledge gap is further confirmed by 94% (n = 47) participants informing us that as a patient they would choose to avoid a drug if they were to be informed that it can impair memory, cause confusion, physical decline, falls with risk of hospital admissions and mortality. Eight percent staff (n = 4) have indicated that they would not avoid these medications despite cognitive side effects, four percent staff (n = 2) were not comfortable to decide on this and two percent staff (n = 1) replied that it depends on the patient. Despite knowing the increased risk of the above morbidity and mortality, two percent (n = 1) staff would not avoid these medications, two percent (n = 1) said they could not comment and two percent (n = 1) said it depends on the situation. There was one participant, an auxiliary nurse, who commented that *“I would still be taking these if I am in pain”*, which highlights the important issue of ‘quality of life’ seen as the biggest priority. Hence the need for high vigilance and judgement required in prescribing to these vulnerable people.

Mirabegron, an oral selective B3-adrenergic receptor agonist, has been an alternative safe drug in terms of anti-cholinergic burden [6]. In our study only 30% knew that it did not have ACB. Twenty-six percent participants were not aware of the burden score of this drug and the rest 44% was of the wrong impression that it could cause anticholinergic burden. This again is an indication of knowledge gap on the issue of ACB. There were six comments received in the free text space and all of them requested for further guidance on this topic of ACB with comments like *“relevant to their practice”,* *“would appreciate teaching on this”, “Interesting topic I was largely unaware of”* and *“need further guidance”.* This snap shot study confirms the need for staff education as well as patient education.

Recent update in 2019 of National Institute for Health and Care Excellence (NICE) guideline NG123, advises to explain to the women the long-term effects of anticholinergic medicines for overactive bladder on cognitive function are uncertain. Including information of anticholinergic burden in the local and national guidelines will help increase awareness of staff and prevent long-term patient morbidity and mortality. Use of aide-memoire in clinical areas helps raise staff awareness. Updated patient information leaflets provided at the time of consultation, with information on cumulative anticholinergic burden will improve patient awareness and long-term outcomes. Regular medication review in primary or secondary care with a view to reduce cumulative anticholinergic burden will avoid continuing these medications for longer than necessary.

Limitations of the study

We included 17 commonly used medications, however, there are many other commonly used medications with high ACB scores, e.g.: solifenacin, sertraline, nortriptyline and prochlorperazine which have not been included. We acknowledge that our numbers are small and we have not particularly concentrated on one group of staff like consultants. We appreciate including nursing and auxiliary staff might skew the results, however, the aim of this study is to identify the level of awareness in all staff groups. We found that the lack of knowledge and awareness was uniform over all health care groups; it may be slightly more in auxiliary and nursing staff group. Our aim was to understand the staff beliefs and attitudes which was served by this study. We believe this is a true representation of the wider community as we have included all genre of staff and the participation is voluntary. The staff were informed that this is anonymous survey of data so as to get the genuine answers. Studies looking at wider group both general population and health care professionals would be able to expand the problems and possible solutions further.

## Conclusions

Anticholinergic burden (ACB) is a serious phenomenon associated with increased morbidity and mortality in the general population as well as elderly population. Despite many studies confirming these life-limiting adverse effects, the awareness among health care staff is limited. *“Primum non nocere”* - “First do no harm” - is the age-old ethical principle governing the practice of medicine. Prescription of anticholinergics to women without adequate counselling about long-term cognitive effects, morbidity and mortality would go against this ethical principle. We believe the main reason for reduced awareness among patients is the lack of awareness of staff who prescribe them. Our study has shown the awareness among all groups of health care professionals is lacking. We appreciate it is hard to measure the adverse effects on different population and measure the benefit of patient education in any one setting of either primary care or secondary care as polypharmacy is usually involved. Long-term follow-up studies to study benefits of patient education and informed consent at the time of prescription of anticholinergics and regular follow-up of patients with a view to assess decline in cognition and general health would be helpful.

## Appendices

**Table 2:** ACB questionnaire ACB: Anticholinergic Burden

Anticholinergic Burden - Awareness amongst health care professionals. A voluntary anonymous survey of staff perception on anticholinergic burden and side-effects. Please can you take a minute to fill this form. Thanks! Please circle your position in the hospital – FY2/ GPST/ Registrar/ Consultant/ Midwife/ Nurse/Auxiliary staff/ Student nurse/ Student Midwife								
Criteria (please mark by a tick in Yes or No box)					Yes			No
Are you aware of the term anticholinergic burden (ACB) If yes- please describe your understanding of ACB								
Do you prescribe anticholinergics in your current role?								
Are you aware of the effect of anticholinergics on cognition (e.g.: memory impairment, confusion)								
Are you aware these effects are marked by a scoring system (anticholinergic burden)?								
If you score the effects of the following drugs on cognition as 0 - 3; none – 0, mild – score 1, moderate – score 2, severe – score 3. What score will you give the following commonly used medications? Please mark (× or tick)								
Drug name	Score 0	Score 1		Score 2			Score 3	
Tramadol								
Cetirizine								
Oxybutynin								
Hydrocortisone								
Chlorpheniramine								
Promethazine								
Amitriptyline								
Trospium								
Fesoterodine								
Prednisolone								
Codeine								
Warfarin								
Nifedipine								
Hydralazine								
Darifenacin								
Tolterodine								
Mirabegron								
Would you be considering to avoid prescribing medications which might affect cognition if possible?								
Do you usually counsel patients on the effect of cognition while starting incontinence medications?								
If you were to be a patient would you like to avoid the medication(s) if you were informed of the specific side effects as below: impaired cognition (memory and confusion), physical decline, risk of falls, hospital admission, increased mortality.								
Please give any comments you feel relevant to this issue. Thank you for completing this.								
